# Supplementary material for: Sex-Specific Effects of Dietary Methionine Restriction on the Intestinal Microbiome
Source: Nutrients. 2020 Mar 16;12(3):781. doi: 10.3390/nu12030781 (PMC7146121; doi:10.3390/nu12030781)
Supplement: Supplementary file 1 [file nutrients-12-00781-s001.zip › nutrients-736856 -Table_S1.pdf]

nutrients

MDPI

Table 1. Nutritional characteristics of the methionine-adequate (TD.140520) and methionine-restricted (TD.160240) diets used in the study.

Formula	TD.140520 g/kg	TD.160240 g/kg
Sucrose	445.297	448.98
Corn Starch	198.783	200
Corn Oil	100	100
Cellulose	30	30
Mineral Mix, AIN-76 (170915)	35	35
Calcium Phosphate, dibasic	3	3
L-Alanine	3.5	3.5
L-Arginine HCl	12.1	12.1
L-Asparagine	6	6
L-Aspartic Acid	3.5	3.5
L-Cystine	3.5	3.5
L-Glutamic Acid	40	40
Glycine	23.3	23.3
L-Histidine HCl, monohydrate	4.5	4.5
L-Isoleucine	8.2	8.2
L-Leucine	11.1	11.1
L-Lysine HCl	18	18
L-Methionine	6.5	1.5
L-Phenylalanine	7.5	7.5
L-Proline	3.5	3.5
L-Serine	3.5	3.5
L-Threonine	8.2	8.2
L-Tryptophan	1.8	1.8
L-Tyrosine	5	5
L-Valine	8.2	8.2
Vitamin Mix, Teklad (40060)	10	10
Ethoxyquin, antioxidant	0.02	0.02
% by weight		
Protein	15.3	16.6
CHO	63.3	63.7
Fat	10	10

*- Vitamin Mix, w/o choline, A, D, E; Vitamin E, DL-alpha tocopheryl acetate (500 IU/g; 0.242 g/kg); Vitamin A Palmitate (500,000 IU/g; 0.0396 g/kg); Vitamin D3, cholecalciferol (500,000 IU/g; 0.0044 g/kg).
