# Supplementary figures and images for: Sex-Specific Effects of Dietary Methionine Restriction on the Intestinal Microbiome
Source: Nutrients. 2020 Mar 16;12(3):781. doi: 10.3390/nu12030781 (PMC7146121; doi:10.3390/nu12030781)

Figure S1: Metabolites in serum

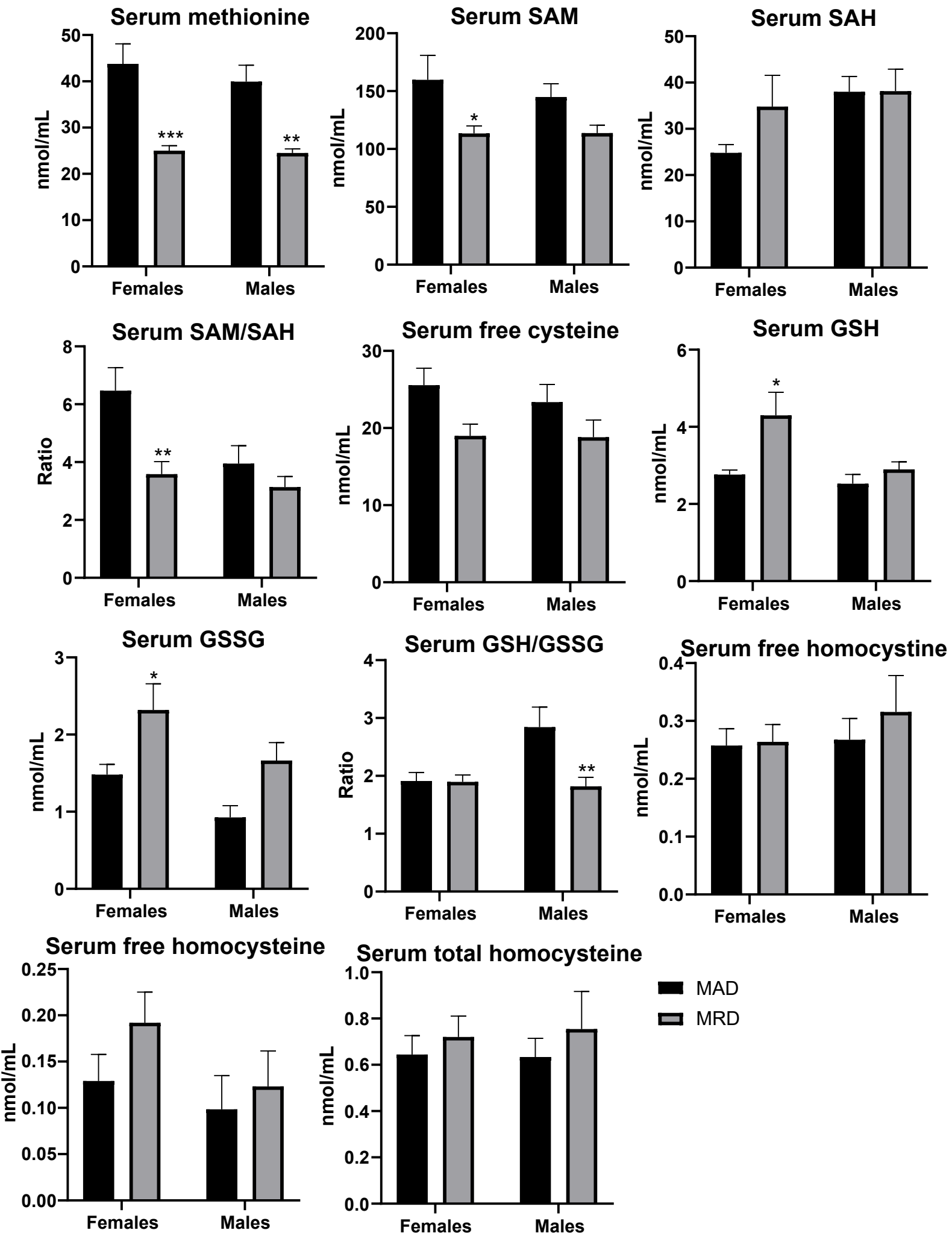

Supplement: Supplementary file 1 [file nutrients-12-00781-s001.zip › nutrients-736856-FigS1.pdf]

Figure S2: Metabolites in liver

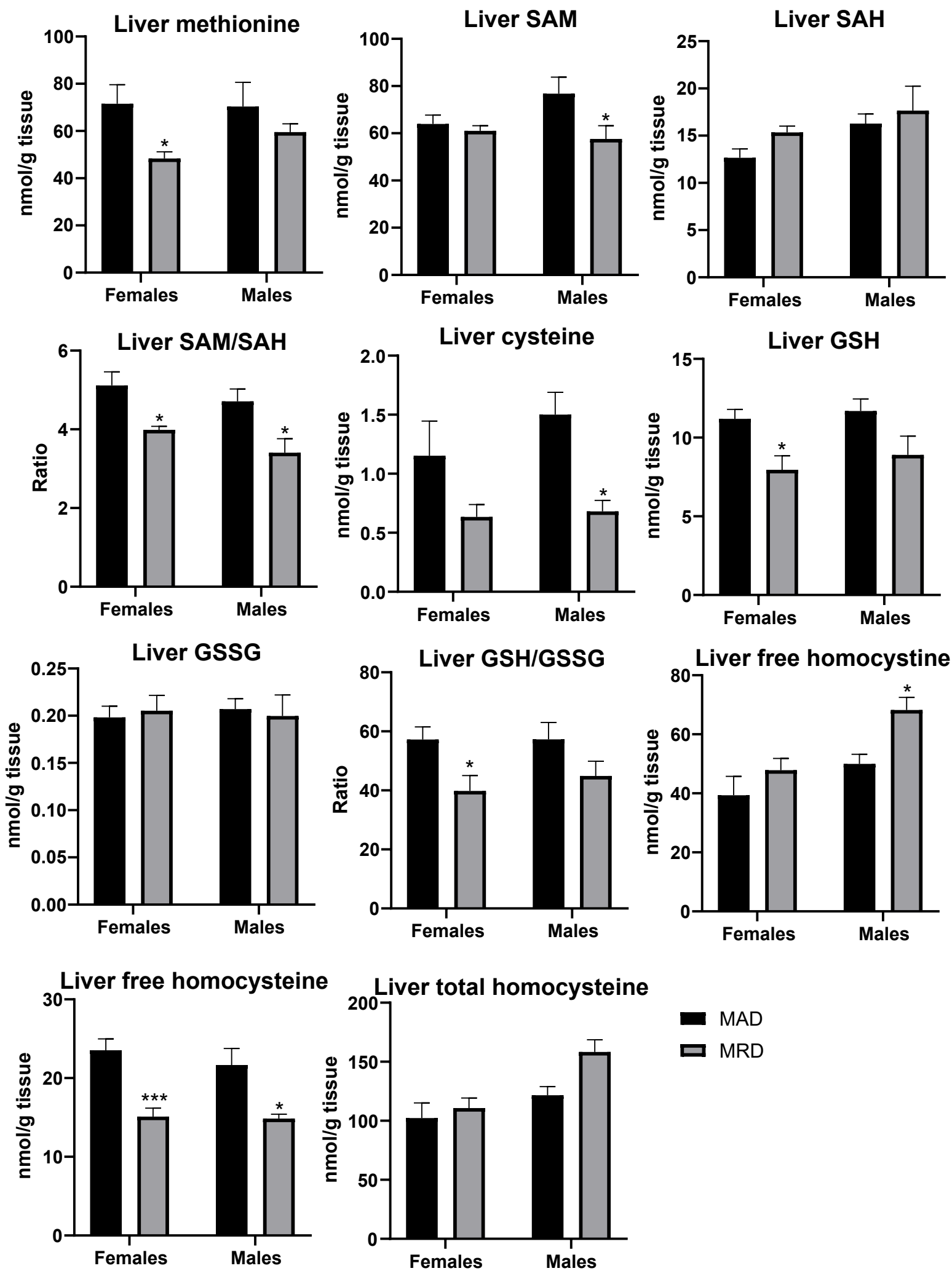

Supplement: Supplementary file 1 [file nutrients-12-00781-s001.zip › nutrients-736856-FigS2.pdf]
